# Cross-protection of commercial vaccines against Chilean swine influenza A virus using the guinea pig model as a surrogate

**DOI:** 10.3389/fvets.2023.1245278

**Published:** 2023-09-20

**Authors:** Rodrigo Tapia, Juan Mena, Victoria García, Marie Culhane, Rafael A. Medina, Victor Neira

**Affiliations:** ^1^Medicina Preventiva Animal, Facultad de Ciencias Veterinarias, Universidad de Chile, Santiago, Chile; ^2^Veterinary Population Medicine, College of Veterinary Medicine, University of Minnesota, St Paul, MN, United States; ^3^Department of Pathology and Experimental Medicine, School of Medicine, Emory University, Atlanta, GA, United States; ^4^Department of Microbiology, Icahn School of Medicine at Mount Sinai, New York, NY, United States; ^5^Departamento de Enfermedades Infecciosas e Inmunología Pediátrica, Escuela de Medicina, Pontificia Universidad Católica de Chile, Santiago, Chile

**Keywords:** influenza, swine, vaccine, cross-protection, guinea pig model, surrogate

## Abstract

Influenza A virus poses a significant threat to public health and the swine industry. Vaccination is the primary measure for controlling the disease, but the effectiveness of vaccines can vary depending on the antigenic match between vaccine strains and circulating strains. In Chile, H1N1pdm09 and other lineages H1N2 and H3N2 have been detected in pigs, which are genetically distinct from the strains included in commercial vaccines. This study aimed to evaluate the cross-protection by commercial vaccines against strains circulating in Chile using the guinea pig model. For this study, four circulating strains [A/swine/Chile/H1A-7/2014(H1N2), A/swine/Chile/H1B-2/2014(H1N2), A/swine/Chile/H1P-12/2015(H1N1), and A/swine/Chile/H3-2/2015(H3N2)] were selected. Guinea pigs were divided into vaccinated and control groups. The vaccinated animals received either a multivalent antigenically heterologous or monovalent homologous vaccine, while the control animals remained unvaccinated. Following vaccination, all animals were intranasally challenged, and nasal wash samples were collected at different time points post-infection. The results showed that the homologous monovalent vaccine-induced hemagglutinin-specific antibodies against the Chilean pandemic H1N1pdm09 strain. However, the commercial heterologous multivalent vaccine failed to induce hemagglutinin-specific antibody titers against the H1N2 and H3N2 challenge strains. Furthermore, the homologous monovalent vaccine significantly reduced the duration of viral shedding and viral titers specifically against the Chilean pandemic H1N1pdm09 strain and heterologous multivalent vaccine only partial. These findings highlight the importance of regularly updating vaccine strains to match the circulating field strains for effective control of swine influenza. Further research is needed to develop vaccines that confer broader protection against diverse strains of swine influenza A virus.

## Introduction

The Influenza A virus (IAV) is widely distributed in pig farms worldwide (1). Outbreaks pose a threat to public health due to their zoonotic potential, and they cause significant economic losses in the swine industry by decreasing productive parameters such as daily weight gain, feed conversion efficiency, and mortality, as well as generating additional expenses for the treatment of sick animals (2).

In Chile, new lineages of H1N2 and H3N2 IAVs have been detected circulating in pigs, which are antigenically distinct from the strains present in the current North American commercial vaccines used in the country. Additionally, a high genetic and antigenic diversity of pandemic H1N1 viruses has been identified. On the other hand, the most used animal models in immunological studies of IAV are the ferret, mouse, and guinea pig. The guinea pig presents several advantages compared to the ferret, making it an attractive model for such studies, including its commercial availability, low cost, small size, and ease of handling and housing. Moreover, the guinea pig is highly susceptible to IAV compared to the mouse, which requires pre-adapted viruses to cause infection (3, 4). Furthermore, the guinea pig model has been approved for safety and efficacy studies in the production of swine influenza vaccines by the United States Code of Federal Regulations (9 CFR 113.200) and the European Pharmacopeia (EUROPEAN PHARMACOPEIA 7.0 01/2008:0963). The objective of this study was to evaluate *in vivo* the protective immune response generated by commercial homologous and heterologous vaccines against field strains present in Chile.

## Materials and methods

### Strains

The strains used for the challenge were IAV-S A/swine/Chile/H1P-12/2014(H1N1), A/swine/Chile/H1A-7/2014(H1N2), A/swine/Chile/H1B-2/2014(H1N2), and A/swine/Chile/H3-2/2015(H3N2), representing genetically and antigenically different clusters previously described in Chile, named: A(H1N1)pdm09-like, Chilean H1 A, Chilean H1 B, and Chilean H3 (1, 5, 6). These viruses corresponded to circulating swine IAV originally derived from humans (5, 7). The strains were grown in Madin-Darby Canine Kidney (MDCK) cells maintained at 37°C with CO_2_. The presence of cytopathic effect (CPE) was evaluated, and once the cell monolayers reached over 70% destruction, the supernatant was harvested and stored at −80°C. HA titers were obtained using the hemagglutination assay described by Kitikoon et al. (8). The viruses were titrated by median tissue culture infective dose (TCID50) assay, expressed in TCID50/mL according to the Reed-Muench method (9). Additionally, the challenge strains were evaluated against vaccine strains reference antisera using HI at Veterinary Diagnostic Lab, College of Veterinary Medicine, University of Minnesota.

The strain A/swine/Chile/H1P-12/2014(H1N1) is antigenically homologous with A/California/04/2009(H1N1). Contrary, the strains A/swine/Chile/H1A-7/2014(H1N2), A/swine/Chile/H1B-2/2014(H1N2), and A/swine/Chile/H3-2/2015(H3N2) do not belong to the lineages presented in the heterologous vaccine (Table 1).

**Table 1:** Results of the HI test of the challenge strains against the commercial vaccine strains reference antisera.

**Strains**	**Reference antisera**				
	**H1** γ **cluster**	**H1** δ**-1 cluster**	**H1** δ**-2 cluster**	**H1pdm09 cluster**	**H3 IV cluster**
A/swine/Chile/H1A-7/2014(H1N2)	<20	<20	<20	-	-
A/swine/Chile/H1B-2/2014(H1N2)	<20	<20	<20	-	-
A/swine/Chile/H1P-12/2014(H1N1)	-	-	-	160	-
A/swine/Chile/H3-2/2015(H3N2)	-	-	-	-	80
H1 γ Cluster	**320**	-	-	-	-
H1 δ-1 Cluster	-	**320**	-	-	-
H1 δ-2 Cluster	-	-	**320**	-	-
H1N1pdm09 Cluster	-	-	-	**320**	-
H3 IV Cluster	-	-	-	-	**320**

The homologous titers are highlighted in bold.

### Experimental design

Pirbright female 4-week-old guinea pigs, provided by the Chilean Institute of Public Health (ISP), were used as pig surrogates. These guinea pigs were influenza-free and acclimated to a 12-h light/dark cycle with *ad libitum* access to food and water for 1 week. Groups of 8 animals each were established for each IAV-S strain to be evaluated: 4 groups were vaccinated, and the other 4 groups were sham-vaccinated as controls. One vaccinated group was vaccinated with the commercial homologous monovalent vaccine [containing A/California/04/2009(H1N1) and Amphigen^®^ as adjuvant] challenged with the pandemic strain A/swine/Chile/H1P-12/2014(H1N1). While the other three vaccinated groups were vaccinated with the commercial heterologous multivalent vaccine, containing strains from the North American H1 clusters γ [A/Swine/Iowa/110600/2000(H1N1), H1 cluster δ A/Swine/Oklahoma/0726H/2008(H1N2), A/Swine/North Carolina/031/2005(H1N1) and H3: IV; A/Swine/Missouri/069/2005(H3N2) and a commercially available oil-in-water emulsion for swine vaccines as an adjuvant], which were challenged with the strains A/swine/Chile/H1A-7/2014(H1N2), A/swine/Chile/H1B-2/2014(H1N2), and A/swine/Chile/H3-2/2015(H3N2) separately. All procedures involving animals were approved by the Institutional Committee for Animal Care and Use (CICUA) of the University of Chile under certificate number 02-2016.

Vaccination was performed through subcutaneous injection of 200 μL at 0- and 14-days post-vaccination (dpv). At 28 dpv, all animals were intranasally challenged with 300 μL of 1 × 10^5^ pfu of each viral strain (10).

### Sample collection and analysis

A 1 mL blood sample was collected from each vaccinated and unvaccinated animal through jugular venipuncture immediately before the challenge. This was done to perform by hemagglutination inhibition (HI) tests to evaluate the hemagglutinin-specific antibody titers in vaccinated animals against the IAV strains used in the challenge and to confirm the absence of antibodies in the unvaccinated animals. It is widely recognized that hemagglutination inhibition antibody titers correlate with neutralizing antibodies and protection against influenza infection (11). For sera inactivation, previous HI assay, 100 μL was incubated at 56°C for 30 min, and then 600 μL of 25% kaolin in borate saline was added and incubated at room temperature (RT) for 30 min. Later, 600 μL of 25% turkey red blood cells were added and incubated at RT for 2 h. The tubes were centrifugated at 400 × g for 5 min, obtaining the inactivated sera as supernatant. For HI, the sera were subjected to serial two-fold dilutions in PBS using bottom V 96 wells plates. Then the virus was added at 8 HAU/50 μL, and incubated at RT for 30 min. Later, 50 μL of 0.5 % Turkey RBC to all wells were added and incubated for 45 min (6, 8). Nasal wash samples were then collected from all guinea pigs on days 1, 3, 5, 7, and 9 post-inoculation. For this, 500 μL of 1 × PBS (supplemented with 0.3% bovine serum albumin and 1 × antibiotic-antimycotic solution) were instilled into each nasal cavity, and the samples were collected in sterile Petri dishes, aliquoted, and stored at −80°C. Prior to sample collection and challenge procedures, the animals were anesthetized with intramuscular ketamine (30 mg/kg) and xylazine (2 mg/kg). After the final sample collection, the animals were euthanized with intraperitoneal sodium thiopental (120 mg/kg). RNA was extracted from each nasal wash sample using TRIzol^®^ LS Reagent (Invitrogen™, Carlsbad, CA, USA) following the manufacturer's protocol. Quantitative reverse transcription-polymerase chain reaction (qRT-PCR) was performed, amplifying a conserved region of the matrix gene (12). Quantification was done using a standard curve, generated with 10-fold serial viral dilutions estimated by pfu and the data were expressed as log10 virus RNA copies/mL, with a limit of detection > 0.25 log10.

### Statistical analysis

The area under the curve (AUC) was calculated to compare the total viral excretion during the sampling period between vaccinated and control groups. Additionally, viral titers (log10 virus/mL) were compared between vaccinated and control groups at each sampling point (post-infection day). The Mann-Whitney *U*-test was performed to determine statistically significant differences between the groups.

## Results

Animals vaccinated with the commercial homologous monovalent vaccine (containing the North American pandemic strain) exhibited hemagglutinin-specific antibody titers against A/swine/Chile/H1P-12/2014(H1N1). In contrast, animals vaccinated with the commercial heterologous multivalent vaccine and control animals were negative for antibodies. The results of HI using the reference vaccine strains antisera showed titers against H1N1pdm09 and H3N2 challenge strains (Table 1).

**Figure 1:**
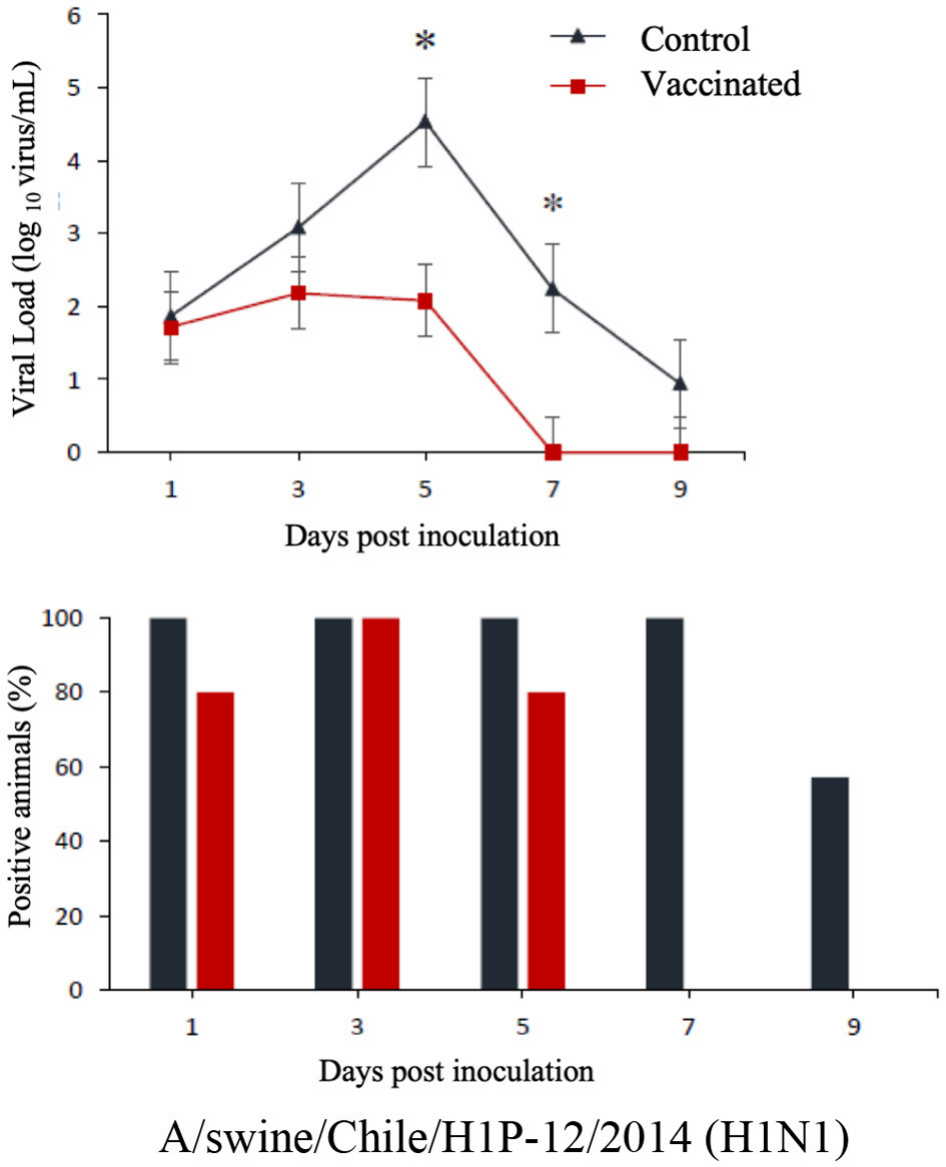
**Top:** Efficacy test of the commercial monovalent vaccine against H1 pandemic 09 virus. Groups of 8 guinea pigs (vaccinated group and its unvaccinated control group) were intranasally challenged with the strain A/swine/Chile/H1P-12/2014(H1N1). Nasal wash samples were taken, and viral load was measured in log10 virus/mL on days 1, 3, 5, 7, and 9 dpi. Asterisks indicate statistically significant differences (*P* < 0.05) between the vaccinated group and the control group. **Bottom:** Percentage of IAV-positive animals per day post-infection in ea ch group.

**Figure 2:**
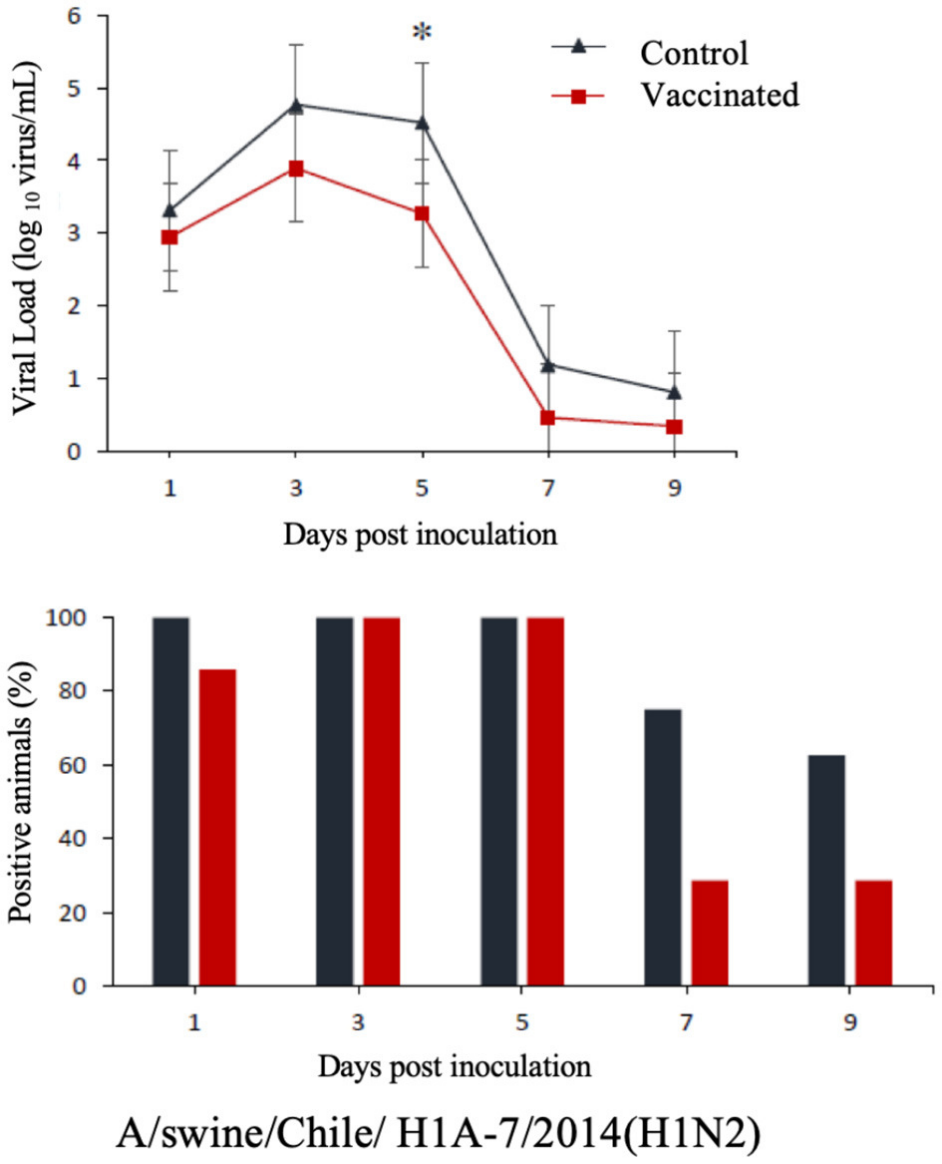
**Top:** Efficacy test of the commercial multivalent vaccine against Chilean H1 virus A. Groups of 8 guinea pigs (vaccinated with the commercial multivalent vaccine and unvaccinated control group) were intranasally challenged with the strain A/swine/Chile/H1A-7/2014(H1N2). Nasal wash samples were taken, and viral load was measured in log10 virus/mL on days 1, 3, 5, 7, and 9 dpi. Asterisks indicate statistically significant differences (*P* < 0.05) between the vaccinated group and the control group. **Bottom:** Percentage of IAV-positive animals per day post-infection in each group.

**Figure 3:**
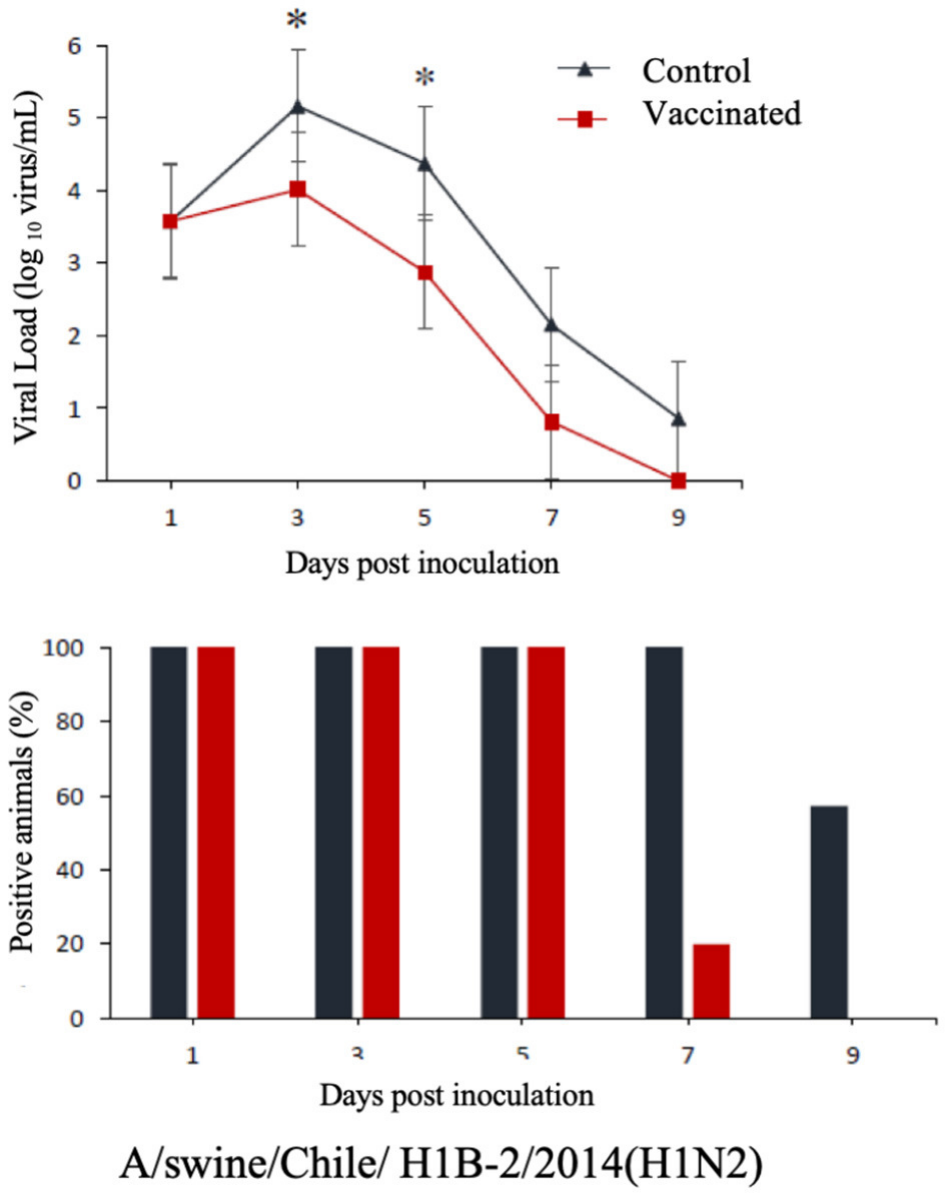
**Top:** Efficacy test of the commercial multivalent vaccine against Chilean H1 virus B. Groups of 8 guinea pigs (vaccinated with the commercial multivalent vaccine and unvaccinated control group) were intranasally challenged with the strain A/swine/Chile/H1B-2/2014(H1N2). Nasal wash samples were taken, and viral load was measured in log10 virus/mL on days 1, 3, 5, 7, and 9 dpi. Asterisks indicate statistically significant differences (*P* < 0.05) between the vaccinated group and the control group. **Bottom:** Percentage of IAV-positive animals per day post-infection in each group.

**Figure 4:**
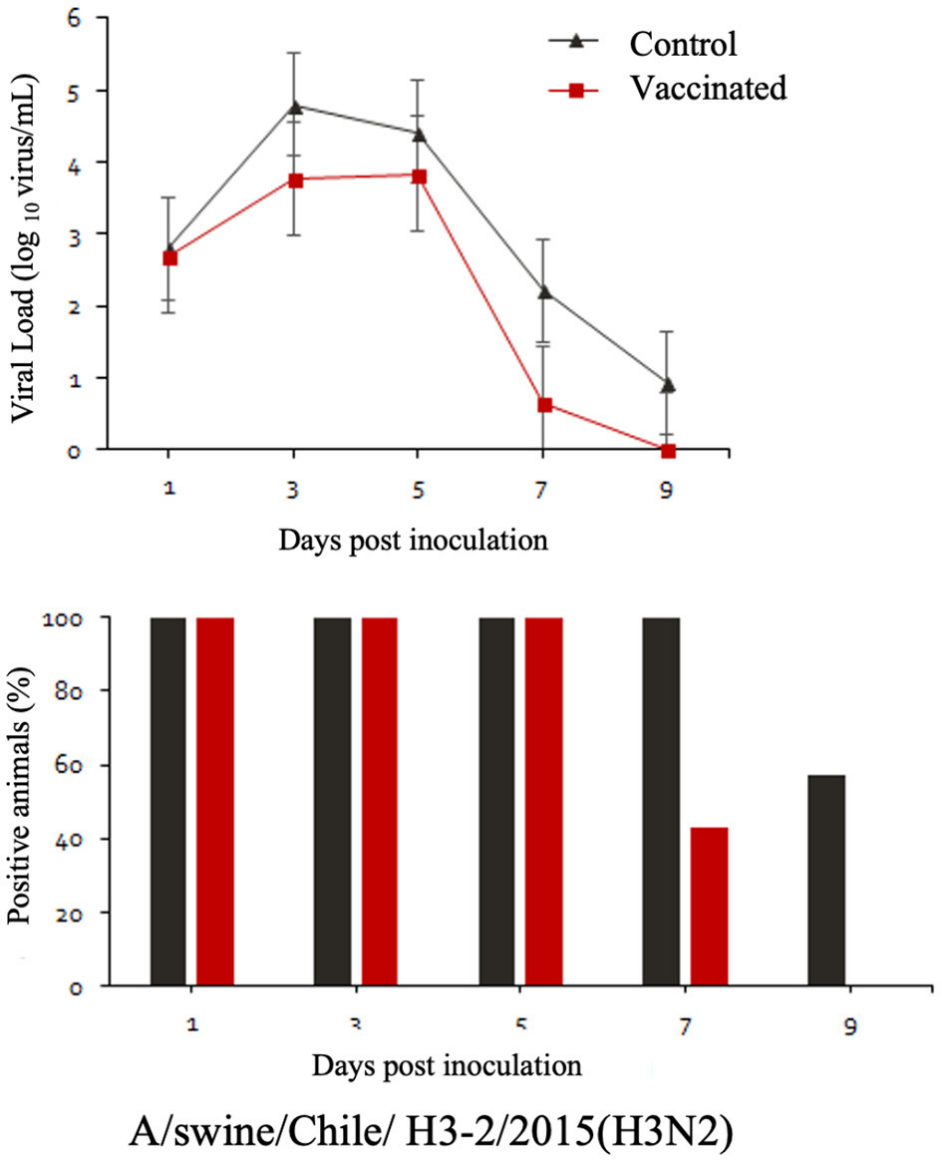
**Top:** Efficacy test of the commercial multivalent vaccine against Chilean H3 virus. Groups of 8 guinea pigs (vaccinated group and their unvaccinated control group) were intranasally challenged with the strain A/swine/Chile/H3-2/2015(H3N2). Nasal wash samples were taken, and viral load was measured in log10 virus/mL on days 1, 3, 5, 7, and 9 dpi. **Bottom:** Percentage of IAV-positive animals per day post-infection in each group.

The commercial homologous monovalent vaccine showed a significant decrease in viral load compared to its control group on days 5 and 7 post-infection when challenged with the pandemic strain A/swine/Chile/H1P-12/2014(H1N1), with a difference of up to 2.4 log10 virus/mL (*P* < 0.05). Positive animals were observed up to day 5 post-infection in the commercial vaccine group, while the control group exhibited positive animals up to day 9 post-infection (Figure 1). On the other hand, animals vaccinated with the multivalent heterologous vaccine demonstrated a slight reduction in viral load compared to their respective control groups (*P* < 0.05) when challenged with the H1 Chilean cluster A, H1 Chilean cluster B, and H3 Chilean strains (Figures 2–4). Overall, animals vaccinated with the multivalent vaccine exhibited an average viral load of 0.8 log10 virus/mL lower than their control group. In detail, in the challenge with the A/swine/Chile/H1A-7/2014(H1N2) strain the commercial multivalent vaccine showed a lower viral load than its control group only on day 5 post-infection, with a difference of 1.2 log10 virus/mL (*P* < 0.05). In both the vaccine and control groups, there were positive animals throughout the experiment, reaching 100% positivity (Figure 2). In the challenge with the A/swine/Chile/H1B-2/2014(H1N2) strain the commercial multivalent vaccine exhibited a lower viral load than its control group on days 3 and 5 post-infection, with a difference of up to 1.4 log10 virus/mL (*P* < 0.05). There were positive animals up to day 7 post-infection, with 100% positivity on days 1, 3, and 5 dpi. In the control group, there were positive animals on all sampling days (Figure 3). Finally, in the challenge with the A/swine/Chile/H3-2/2015(H3N2) strain, the viral loads were not statistically different between animals vaccinated with the commercial multivalent vaccine and the control group. In the commercial vaccine group, there were positive animals up to day 7 post-infection, with 100% positivity on days 1, 3, and 5 dpi. In the control group, there were positive animals until day 9 (Figure 4).

## Discussion

Influenza A virus (IAV) is a significant threat to both public health and the swine industry worldwide. Vaccination plays a crucial role in controlling the disease; however, the effectiveness of vaccines can vary depending on the antigenic match between vaccine strains and circulating field strains. This study aimed to evaluate the cross-protection provided by commercial vaccines against field strains circulating in Chile. The results of this study demonstrated that the commercial vaccines exhibited different levels of cross-protectivity against the Chilean field strains of swine influenza A virus. The homologous monovalent vaccine, which contained the North American pandemic strain, induced hemagglutinin-specific antibodies against the Chilean pandemic H1N1pdm09 strain. This finding suggests that the monovalent vaccine was effective in providing protection specifically against the closely related strain. In contrast, the commercial heterologous multivalent vaccine, which contained strains from the North American H1 clusters γ, δ, and H3 cluster: IV, did not induce specificantibody titers against the H1N2 and H3N2 challenge strains. This lack of cross-reactivity suggested that the heterologous multivalent vaccine may not offer sufficient protection against these genetically distinct field strains circulating in Chile. Furthermore, the efficacy of the vaccines was assessed based on viral shedding and viral titers in the nasal wash samples. The homologous monovalent vaccine significantly reduced the duration of shedding and viral titers against the Chilean pandemic H1N1pdm09 strain, indicating its ability to control viral replication and limit the spread of the virus. In contrast, the heterologous multivalent vaccine only demonstrated a partial reduction in viral shedding on specific days post-infection for the H1N2 and H3N2 strains. However, no significant differences were observed in viral shedding between the vaccinated and control groups for the H3N2 strain.

The strains from the Chilean H1 cluster A, Chilean H1 cluster B, and Chilean H3 cluster are genetically divergent from all known influenza A viruses worldwide to date (1, 6). It has been widely reported that currently used inactivated commercial vaccines are effective only against genetically and antigenically related viruses (13, 14). Therefore, the development and use of vaccines with strains circulating in a specific geographical region are essential for efficient disease control (15). It is worth noting that the heterologous multivalent vaccine was able to slightly reduce viral shedding of the Chilean H1 cluster A and B viruses, even though vaccinated animals did not have hemagglutinin-specific antibodies (directed against HA) prior to the challenge (Supplementary Figure 1). This could be attributed to the production of antibodies against NA induced by the vaccine, which inhibits the enzymatic activity of this glycoprotein, preventing the release of new viral particles from infected cells. Previous studies have shown that in the absence of neutralizing antibodies, NA antibodies can provide varying degrees of protection against the disease due to the presence of conserved epitopes (16). These results support the importance of NA in vaccine-induced protection (17) and emphasize the need for genetic and antigenic characterization of NA in these new IAVs.

These results suggest that the heterologous multivalent vaccine provided limited protection against the H1N2 and H3N2 challenge strains, possibly due to the antigenic mismatch between the vaccine strains and the circulating field strains. We indicate “partial or limited” protection instead of low or moderate protection because all animals were infected at some point in the challenge. These findings emphasize the importance of regularly updating vaccine strains to match the antigenic characteristics of circulating field strains for effective control of swine influenza. The genetic and antigenic diversity of the influenza viruses circulating in pigs, as observed in Chile, highlights the need for continuous surveillance and strain selection to ensure the development and implementation of vaccines that confer broader protection against diverse strains of swine influenza A virus.

However, it is important to acknowledge several limitations of this study. Firstly, the evaluation of vaccine effectiveness was conducted using the guinea pig model, which may not fully reflect the immune response and protection conferred in swine. As mentioned before, the guinea pig model is advantageous due to lower costs, ease of working, and implementation in smaller facilities, which could benefit the study of swine IAV in developing countries. The guinea pig model has been approved for safety and efficacy studies in the production of swine influenza vaccines, which typically involves determining the viral titers at 24 h and 72 h after the challenge. In this study, evidence of the viral dynamic includes the viral clearance after challenge instead of the validation of commercial vaccines. Although guinea pigs are commonly used as a surrogate model for studying influenza, there can be differences in immune responses and viral pathogenesis compared to the natural host. Remarkably, guinea pigs are infected and are able to transmit influenza viruses without any clinical signs (18).

Therefore, caution should be exercised when extrapolating these findings to swine populations. However, in this challenge using homologous and heterologous vaccines, the results are consistent even though the vaccines were originally prepared for swine. Similar results have been obtained in similar settings using pigs (19).

Additionally, this study solely assessed the humoral immune response in terms of neutralizing antibody titers and viral shedding. While hemagglutinin-specific antibodies is an important correlate of protection, other components of the immune response, such as cellular immunity, may also contribute to vaccine effectiveness. Therefore, the impact of cellular immune responses on cross-protection was not investigated in this study and warrants further investigation.

In conclusion, this study provides valuable insights into the cross-protection provided by commercial vaccines against field strains of swine influenza A virus in Chile. The results indicate that the commercial vaccines had varying levels of efficacy against the circulating field strains, with the homologous monovalent vaccine demonstrating better protection compared to the heterologous multivalent vaccine. These findings underscore the importance of regularly updating vaccine strains to match the antigenic characteristics of circulating field strains, ultimately contributing to more effective control strategies for swine influenza. Further research is needed to develop vaccines that confer broader protection against diverse strains of swine influenza A virus, considering the genetic and antigenic diversity observed in different geographical regions.

## Data availability statement

The original contributions presented in the study are included in the article/Supplementary material, further inquiries can be directed to the corresponding author.

## Ethics statement

All procedures involving animals were approved by the Institutional Committee for Animal Care and Use (CICUA) of the University of Chile under Certificate Number 02-2016. The study was conducted in accordance with the local legislation and institutional requirements.

## Author contributions

RT, RM, and VN contributed to the conception and design of the study. RT, JM, VG, MC, and VN performed the analysis. RT and VN wrote the first draft of the manuscript. All authors contributed to the manuscript revision and approved the submitted version.
