# Supplementary material for: Cross-protection of commercial vaccines against Chilean swine influenza A virus using the guinea pig model as a surrogate
Source: Front Vet Sci. 2023 Sep 20;10:1245278. doi: 10.3389/fvets.2023.1245278 (PMC10548122; doi:10.3389/fvets.2023.1245278)

Supplementary information

Figure 1. Title of HI neutralizing antibodies from animals vaccinated against IAV-S challenge strains. Titers of neutralizing antibodies against the strains A/swine/Chile/H1A-7/2014(H1N2) (H1A), A/swine/Chile/H1B-2/2014(H1N2) (H1B), A/swine/Chile/H1P-12/2014(H1N1) (H1P), and A/swine/Chile/H3-2/2015(H3N2) (H3), measured at 28 dpv (prior to challenge) using the HI test. The animals were vaccinated with the experimental multivalent vaccines, only the homologous commercial vaccine did not have antibody titers (HI titer >20).

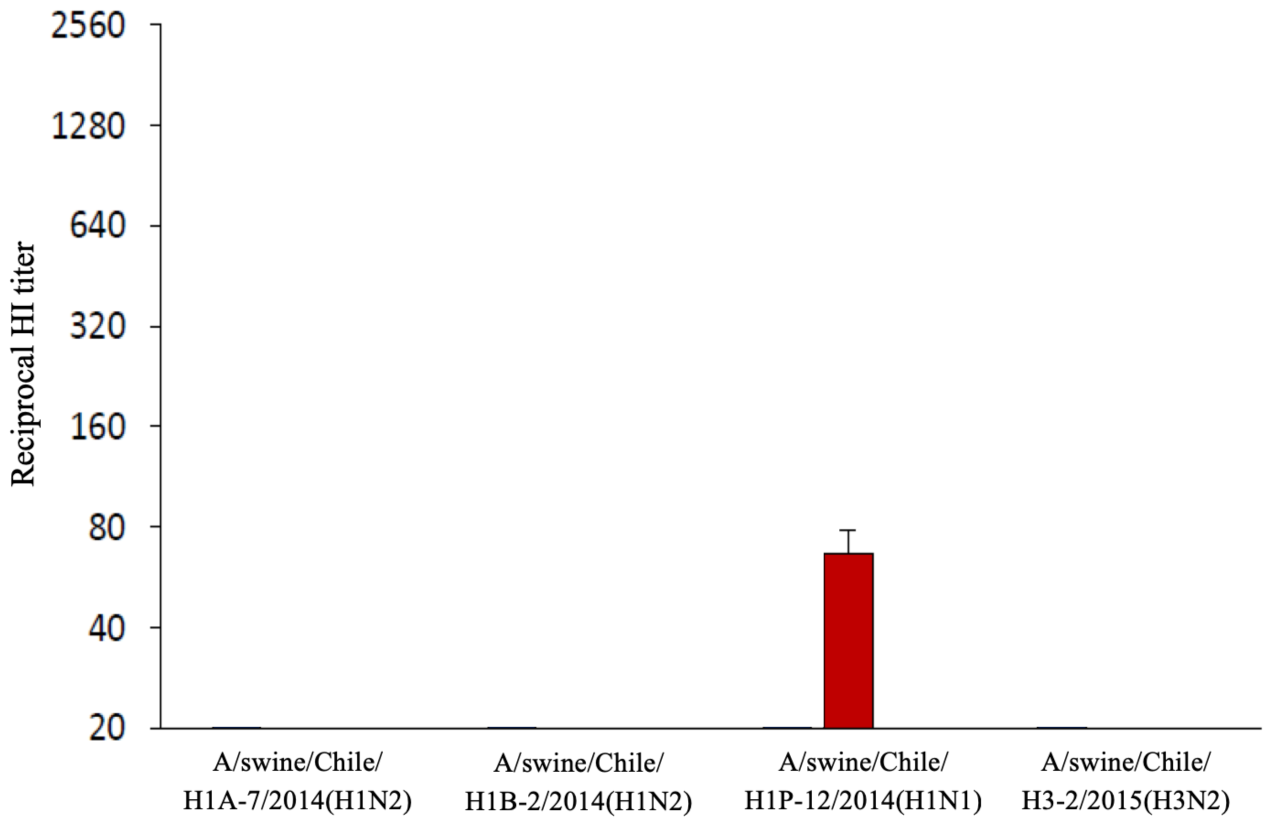

Supplement: Supplementary file 1 [file Image_1.pdf]
